# Impact of fertility on the longevity of older rural Chinese women: an analysis of a longitudinal survey

**DOI:** 10.1186/s12889-022-13039-6

**Published:** 2022-04-07

**Authors:** Wan-li Zhou, Shuo Zhang, Hua-lei Yang, Ying-wen Gu, Yi-dan Yao, Yuan-yang Wu, Si-qing Zhang

**Affiliations:** 1 School of Finance and Public Administration, Hubei University of Economics, 8 Yangqiaohu Avenue, Canglong Island Development Zone, Wuhan, China; 2 School of Public Administration, Zhongnan University of Economics and Law, 182 Nanhu Avenue, Donghu New Technology Development Zone, Wuhan, China

**Keywords:** Number of children, Birth structure, Rural women, Older adults, Longevity

## Abstract

**Background:**

This study evaluates the impact of fertility during the childbearing period on the longevity of older rural Chinese women and verifies whether any trade-off exists between women’s longevity and their number of children to provide empirical evidence for improving health intervention policies and formulating active fertility policies in low-fertility countries.

**Methods:**

Based on the data of the deaths of 1623 older adults aged 65 and above during 2014–2018 in the Chinese Longitudinal Healthy Longevity Survey, this study explores the relationship between the number of children born and older rural women’s longevity using the ordinary least squares method. Furthermore, the impact of fertility on the longevity of men and women in rural and urban areas, along with other reproductive behaviours on older rural women’s longevity, were analysed.

**Results:**

There was a significant negative correlation between the number of children born and women’s longevity (β = − 0.555, *p* < 0.05). Additionally, their longevity exhibited a decreasing trend with having birthed more sons and an increasing trend with more daughters. Age at first and last births had a significant positive relationship with rural women’s longevity; however, the effect of fertility on the longevity of older rural and urban men and older urban women was not significant.

**Conclusions:**

It is confirmed that there is a trade-off between fertility and longevity for rural women in China. Future research should focus on compensating for the decline in female longevity caused by the number of children born and promote the concept of a healthy pregnancy, scientific nurture, and gender equality in fertility.

## Background

The trade-off between fertility and human longevity has attracted much scholarly attention. For example, evolutionary theory suggests that individual organisms live longer by reducing the resources devoted to reproduction [1]. Disposable soma theory (DST) suggests that reproductive investment at the expense of somatic maintenance leads to ageing because organisms have limited resources. In addition to the priority for growth, the remaining resources will be allocated between maintaining and repairing the body and reproduction. Maintenance and repair can counteract body damage and slow down ageing caused by accumulated molecular and somatic cell damage. However, fertility consumes a large proportion of resources, resulting in the reduction of resources used to maintain and repair body cells and leading to a loss of longevity. There is a trade-off between fertility and longevity; increased longevity comes at the cost of reduced fertility, while increased fertility leads to a shorter lifespan [2–5]. DST postulates that the negative correlation between female fertility and lifespan can be understood as an evolutionary trade-off between reproduction and survival [6] known as the fertility-longevity trade-off hypothesis. The effect of fertility on longevity is not only through so-called biological or physiological channels but also via socio-economic factors that regulate the trade-off between fertility and longevity.

China has always followed the concept of ‘more children, more blessings’. It is believed that the more children one has, the more care services, financial support, and emotional comfort he will get from those children when he is in old age. In this way, the number of children is directly related to the quality of life and well-being of parents in their later years and will have a positive impact on the health of older adults and promote life extension [7, 8]. Against such a unique social and cultural background and coupled with the impact of China’s fertility, healthy ageing, and other policies, it is important to explore what kind of impact fertility has on the longevity of older adults in rural China, especially on the life span of rural older women, and whether there is a trade-off relationship between fertility and longevity.

Exploring these questions can further prove the applicability of the fertility–longevity trade-off hypothesis to the Chinese population, expand the explanatory scope of the theory, and promote its development; simultaneously, it can improve health intervention policies and provide empirical evidence for the formulation of active fertility policies in low-fertility countries, with practical implications for promoting health and reducing poverty in high-fertility, developing countries with a low population of older adults.

### Literature review

In response to the above questions, we will review the existing research in three dimensions: the number of births, gender differences in fertility, and the impact of other reproductive behaviours on the life span of older adult women to highlight our research contribution and significance.

The trade-off between fertility and longevity remains controversial. Using historical data from the British aristocracy, Westendorp and Kirkwood [9] found that women who lived the longest had fewer children. Almost half the women who lived to 80 years and above had no children. They also found a similar result for men. After controlling for the effects of differences in health and mortality selection, the trade-off between fertility and longevity still exists for women but not for men [10]. While these findings have been criticised, especially in terms of data quality, statistical analysis methods, and conclusions [11, 12], they sparked interest in the relationship between fertility and longevity [13]. Despite much subsequent research using historical and cohort data, there is insufficient evidence supporting the existence of a trade-off between fertility and longevity in humans, and there are no consistent conclusions about the relationship [14–16].

Some studies using national-level and micro-level individual data demonstrate the existence of the trade-off between fertility and longevity. Research using national-level data has mostly used the average number of children as an indicator to measure fertility and life expectancy to measure the life expectancy of different age groups, after controlling for historical, religious, geographical, socio-economic and parasitic factors. The results have shown that there is a significant negative correlation between the average life expectancy and the average number of children [17]. At the same time, research using micro-level data used the actual number of children as an indicator of fertility. Using the Cox proportional hazards model, Dribe [5] estimated the effect of the number of children on mortality in old age based on historical micro-level data from southern Sweden and found that the number of children had a significant negative effect on longevity for women aged over 50 years, but not for men. For landless women, a higher number of children was linked to a higher mortality rate, suggesting that socio-economic factors are the main channels for the negative impact of fertility on life span rather than the so-called biological or physiological channels. Kuningas et al. [18] studied the relationship between fertility, age at menarche, age at onset of menopause, and mortality in 3575 married women in Rotterdam using the number of children born to measure fertility. There was no correlation between fertility, age at menarche, and onset of menopause, but fertility was related to mortality. The mortality of women with two to three children was lower than those without or with four or more children. Using data from the Krummhörn region of north-western Germany from 1720 to 1870, Lycett et al. [4] found that married women without children lived longer than those with children, and women who had only one child lived longer than those with many children.

Certain scholars believe that the trade-off relationship between the number of births and longevity does not exist or that the two factors are positively correlated [4, 19–21]. Using the data of 6359 women born in the Netherlands (1850–1910), Kaptijn et al. [6] found no trade-off between fertility and life span during the epidemic transition period. Using rural data from New South Wales, Australia, Simons et al. [16] found that the mortality of women with no children increased in their old age and that the mortality of women with children decreased gradually with an increase in parity. Lockhart, Martin, Johnson, Shirtcliff, and Poon [22] studied the effect of the total number of children on life span in 197 female centenarians in Georgia, USA, and found the total number of children born and life span to be positively correlated. However, when smoking and other unhealthy lifestyle practices are considered, the correlation between the total number of children and longevity weakens. Adult children may provide social and economic support for their middle-aged and older adult parents, which is conducive to extending their parents’ life span, and the impacts on the longevity of the father and the mother differ [23]. Chereji, Gatz, Pedersen, and Prescott [24] used the data of 15,622 pairs of twins born in Sweden from 1901 to 1925 to test the relationship between fertility and life span. The survival rate of women and men with children was significantly higher than that of women and men without children. The impact of having children on the survival rate of men was greater because men with children could receive more intergenerational support in their later years [25]. Li and Zhang’s [26] log-logistic study used data from the 2002–2012 Chinese Longitudinal Healthy Longevity Survey (CLHLS) to show that a younger age at first birth reduced women’s survival rate in old age, while women who had more than five children survived longer. It may be that the more children they have, the more likely they are to get support from them and the more satisfaction they get from ‘more children, more happiness’ [27, 28]. The positive relationship between fertility and longevity may be tied to late births [29].

In terms of child gender differences, most studies have suggested that having daughters can significantly improve the life span of older women, but the effect of having sons is controversial. For example, based on a sample of 375 Sami women over 50 years of age from northern Finland before industrialisation, Helle, Lummaa, and Jokela [30] found that the life span of a mother decreased by 0.65 years for each son while increasing by 0.44 years for each daughter. Harrell, Smith, and Mineau [31] further found a significant correlation between children’s gender and mothers’ life expectancy. Jasienska, Nenko, and Jasienski [19] found that the number of daughters and sons had a negative impact on the life span of mothers, based on data from rural Poland; for every additional son or daughter, a mother’s life span would be reduced by 95 weeks. Zeng, Brasher, Gu, and Vaupel [32] used CLHLS data and found that having daughters reduced the risk of death in older adults, in contrast to sons. Compared to older adults aged 65–79, the impact on those aged 80 and older was more significant. Compared to older adults in urban areas, the impact on older adults in rural areas was more significant.

Beise, Voland, Helle, Lummaa, and Jokela [33] did not find a negative correlation between the number of sons born and maternal longevity in pre-industrial population samples from Canada and Germany. While Van de Putte, Matthijs, and Vlietinck [34] found a negative correlation between the number of sons born and maternal longevity in a sample born in rural Flanders, Belgium, between 1700 and 1870, the findings were limited to women born before 1815 who had married ‘common labourers’. Cesarini, Lindqvist, and Wallace [35] found no evidence of an adverse effect of having sons on maternal longevity based on a sample of 900 pre-industrial Sami women from northern Sweden. Pham-Kanter and Goldman [36] used data from the CLHLS and the Taiwan Longitudinal Study of Ageing to estimate the relationship between child gender composition and parental mortality with the Cox proportional hazards model. They found that having sons had no effect on reducing parent mortality in mainland China and Taiwan; however, having daughters had a significant impact on lowering parent mortality. This is because daughters provide more emotional and intergenerational support to their parents.

In terms of the impact of other fertility behaviours on the life span of older women, most studies have suggested that having children later in life is beneficial to women’s longevity, but there is debate regarding the effect of age at first birth. For example, Westendorp and Kirkwood [9] found that women who died early had the lowest ages at first birth, while those who died at the oldest ages had the highest ages at the birth of the first child. Dribe [5] estimated the effect of the time of the first and last birth on old-age mortality and found that the age at first birth had no significant effect on old-age mortality. In contrast, age at the last birth had a negative effect on old-age mortality, particularly before the age of 30. Fuster [37] used data from 1502 inhabitants of Los Nogales in Spain, in 1877–1899, to study the impact of birth history on maternal longevity, including age at first and last birth and the ratio of the number of live births to the number of surviving children. They found that mothers with lower numbers of children that died before the age of 15 lived longer; age at first birth had no significant effect on the life expectancy of women over 50 years. This finding is consistent with Lockhart, Martin, Johnson, Shirtcliff, and Poon [22].

It is evident that few studies have examined the relationship between the number of births and longevity among older women in rural China [38]. Whether the trade-off between fertility and life span also exists in rural Chinese women needs to be further verified. In terms of variable selection, most studies use mortality and life expectancy to measure longevity, but mortality and life expectancy are not a person’s real longevity, which affects the internal validity of the research. In addition, the extant research also ignores the differences between urban and rural areas and gender in the relationship between fertility and longevity. Using the existing literature as a foundation, and based on the CLHLS from 2014 to 2018, we examine the effects of the number of children, gender differences in children born, and other reproductive behaviours (the age of the first and last births) on the longevity of older rural women in China. Furthermore, we analyse the urban-rural heterogeneity of the relationship between fertility and longevity.

## Methods

### Data

We used 2014–2018 CLHLS data that was jointly organised by the Research Centre for Healthy Ageing and Development of Peking University and Duke University. The survey covers 22 representative provinces/cities/autonomous regions in eastern, central, and western China, accounting for about 85% of the total population. About half of the cities/counties were randomly selected as research points for investigation. The survey started in 1998, using a multi-stage stratified sampling method to conduct a household survey. The older adult group, aged 65 and above, were interviewed once every two years. By 2018, the survey had been conducted eight times. The content covers demographic and sociological characteristics, economic status, fertility status, chronic diseases, family income, time of death, and age at death, among others. The CLHLS dataset is the largest and most comprehensive micro panel dataset about older adults in China. It has good reliability and validity and provides valuable data for the study regarding the impact of the number of children born in rural families on women’s longevity.

During the two CLHLS surveys in 2014 and 2018, 2226 older adults aged 65 and above died. We screened the participants who had partaken in the 2014 survey but died before the start of the 2018 survey and obtained the information of the older adults before and after death by matching the datasets of the elderly survival period and death information. After eliminating the missing values of key variables, there were 2145 remaining samples, including 1623 rural samples (834 women and 693 men) and 522 urban samples (306 women and 216 men)^1^.

### Variables

#### Longevity

Longevity is the dependent variable in this study. Referring to the practice of Mittledorf [39], we use the age of the older adult at the time of death to express longevity.

#### Fertility

The core explanatory variables of this study are the number of children born, number of sons born, and number of daughters born. In the dataset of the surviving older adults, the number of children ever born, which mainly refers to the sum of the number of sons and daughters born by the older adults, was recorded. In addition, to continue studying the impact of gender differences and other reproductive behaviours on longevity, this study also selected whether the individual had a son, a daughter, or both sons and daughters; age at first birth; and age at last birth as explanatory variables.

In order to identify the causal effect of the number of children on the longevity of rural older women, it is necessary to solve the endogenous problem between the number of children and the longevity of rural older women. We used the gender of the first child as the instrumental variable of the number of children to identify the causal effect of the number of children on the longevity of rural older women. If the first child is a boy, the variable is assigned as 1; if the first child is a girl, the value is 0. The number of children of our samples with one or more accounted for 99.16%, this implies that the instrumental variables meet the inclusion criterion.

#### Other covariates

In this study, we controlled the variables that affect both fertility behaviour and longevity from three aspects: individual characteristics, family characteristics, and regional factors, to reduce the estimation bias caused by omitted variables as much as possible. Individual characteristics include whether the participants are ethnic minorities, have received a formal education, have chronic diseases, participate in social medical insurance and social endowment insurance, or smoke. Family characteristics include marital status, living arrangements, and whether the kitchen uses clean fuel. The regional factor is based on the provinces of the respondents.

Education level is one of the important indicators to measure social status, and it not only affects the number of births [40] but is also closely related to the age of the first birth [41]. Chronic diseases have become the main cause of premature death among Chinese residents [42]. We used chronic diseases to measure the health status of the older adult before death. We selected hypertension, diabetes, heart disease, stroke, respiratory diseases, and cancer to evaluate the participants’ health status. Medical insurance can reduce the price of medical services borne by older adults, promote timely medical treatment, and improve an individual’s health status. We recognised any one of the basic medical insurance options for urban employees (basic medical insurance for urban residents, new rural cooperative medical insurance, public medical insurance, and commercial medical insurance) as participating in medical insurance. Endowment insurance is a significant source of income for older adults and is an important indicator to measure their economic status. Considering that lifestyle is also an important factor affecting the health of older adults, we considered smoking as a control variable.

We divided the marital status of the older adults into two categories: with spouse and without spouse. We regarded living with a spouse, children (including grandchildren), and other relatives as living with family members and living in pension institutions as not living with family members. Indoor air is also an important factor affecting the health of older adults [43]. We evaluated whether clean fuel was used in the kitchen to measure the indoor environment of the family. Natural gas, gas, sun, and other pipeline-based energy were identified as clean fuel, and kerosene, coal, charcoal, and firewood were identified as non-clean fuel.

The relationship between longevity and fertility may be regulated by the level of socio-economic development and cultural customs among regions [17]. To reduce the estimation bias caused by regional differences, we divided the 22 provinces in CLHLS into eastern, central, and western regions according to China’s regional division rules and assigned them values of 1, 2 and 3, respectively. The eastern region includes Liaoning, Hebei, Beijing, Tianjin, Shanghai, Jiangsu, Zhejiang, Fujian, Shandong, Guangdong, and Guangxi; the central region includes Jilin, Heilongjiang, Shanxi, Anhui, Jiangxi, Henan, Hunan, and Hubei; the western region includes Shaanxi, Sichuan, and Chongqing. The definition and descriptive statistics of each variable are shown in Table 1.

**Table 1:** Variable definition and descriptive statistics (*n* = 2145)

Variable	Definition	Rural Sample (*n* = 1623)			City Sample (*n* = 522)		
		Mean	Percent	SD	Mean	Percent	SD
Longevity	Age of the older adult at the time of death	93.41		9.18	91.76		10.01
Number of children	Total number of children	3.67		1.84	3.50		1.64
Number of sons	Total number of sons	2.42		1.45	2.36		1.34
Number of daughters	Total number of daughters	2.14		1.56	1.93		1.39
Having a son	Whether the number of sons is greater than 0; if yes, it is assigned 1; otherwise, it is 0		94	0.22		97	0.17
Having a daughter	Whether the number of daughters is greater than 0; if yes, it is assigned 1; otherwise, it is 0		87	0.33		89	0.31
Having sons and daughters	If the older adult has at least one son and one daughter, the value is 1; otherwise, it is 0		84	0.37		86	0.34
Age at first birth	The age when the first child was born	24.1		5.71	24.21		4.43
Age at last birth	The age at which the youngest child was born	37.42		6.89	35.67		6.11
Marital status	With spouse assigned 1, and without spouse assigned 0	0.76	76	0.42		68	0.47
Ethnic group	Han nationality is assigned 0, the Hui, Mongolian, and other nationalities as minorities are assigned 1		16	0.37		6	0.23
Education status	Formal education is assigned 1, otherwise, it is 0		35	0.48		61	0.48
Living with family	Whether living alone, if yes, it is 0; if no, it is 1		71	0.45		64	0.48
Hypertension	If the participant suffers from this disease, it is assigned 1; if no, it is 0		28	0.45		37	0.48
Diabetes	If the participant suffers from this disease, it is assigned 1; if no, it is 0		5	0.22		6	0.24
Cardiovascular disease	If the participant suffers from this disease, it is assigned 1; if no, it is 0		20	0.40		26	0.44
Apoplexy	If the participant suffers from this disease, it is assigned 1; if no, it is 0		13	0.34		20	0.41
Respiratory disease	If the participant suffers from this disease, it is assigned 1; if no, it is 0		13	0.34		19	0.39
Cancer	If the participant suffers from this disease, it is assigned 1; if no, it is 0		10	0.30		19	0.39
Medical insurance	If the participant participates in one of the insurances, the value is assigned 1; the value is 0 otherwise		90	0.30		86	0.34
Public older adult insurance	If the participant participates in public older adult insurance, the value is assigned 1; the value is 0 otherwise		23	0.42		5	0.35
Smoking	If the participant ever smokes, the value is assigned 1; otherwise, the value is assigned 0		7	0.25		1	0.08
Clean energy in the kitchen	If the participant uses clean energy, the value is assigned 1; otherwise, it is assigned 0		53	0.50		74	0.43

*Note*: The binary variables only report the proportion of samples with a value of 1

### Statistical model

Considering that the longevity (age at death) of rural older adults is a continuous variable, it basically satisfies the assumption of normal distribution^2^. This study used the ordinary least squares (OLS) regression model to estimate the parameters. The general expression of the model is as follows:

1 $${L}_i=\upalpha +{\beta}_1{FB}_i+\delta {X}_i+{\varepsilon}_i$$

where *L*_*i*_ refers to the longevity of older adults in rural areas; *FB*_*i*_ refers to fertility behaviour, including the number of children, number of sons, number of daughters, whether they have sons, whether they have daughters, age at first birth, and age at last birth; *X*_*i*_ is the control variable; *ε*_*i*_ is the error term; *β*_1_ is the parameter to be estimated, and it reflects the direction and degree of the impact of reproductive behaviour on rural older adults’ longevity; and α is a constant term.

The main problem faced by model (1) is the endogeneity of explanatory variables. The reason for the endogeneity of the model is the omitted variables, such as genetics, health level, and other unobservable variables, which will affect both the fertility and longevity of the older adults; this results in a problem of omitted variables. Therefore, without controlling for the endogeneity of the model, the OLS estimation results will lead to estimation bias.

The instrumental variable method is a common method to overcome the endogeneity of a model. Effective instrumental variables need to meet two conditions. One is related conditions, that is, instrumental variables must be highly correlated with endogenous variables. The second condition is the exclusion condition, that is, the instrumental variable must be exogenous and not related to the error term. We selected the gender of the first child as the instrumental variable for the number of children. The gender of the first child meets two basic conditions for becoming an effective instrumental variable: first, because the gender of the first child is usually random [44]. Although there has always been a phenomenon of ‘son preference’ in rural areas of China, some parents choose the child’s gender through foetal gender identification, resulting in the non-randomness of the child’s gender composition in the family. However, the objects of our analysis are the rural elderly over 65 years old. Most of their childbearing time was concentrated in the 1970s and before. At that time, foetal gender identification technology was not popularised in rural areas of China, rural parents are less likely to choose their children’s gender through foetal gender identification, especially since the implementation of the family planning policy in 1979, the law stipulates that a family can only have one child. In addition, China’s family planning policy was implemented in 1979, and the sample reproductive behaviour has not been limited by family planning policy. The gender of the first child is exogenous [45], which is not related to the residual^3^; Second, men’s reproductive preference directly affects the number of children. Parents whose first child is a boy will stop giving birth earlier than parents whose first child is a girl. Compared with parents whose first child is a daughter, parents whose first child is a son will have fewer children, and the proportion of sons will be higher [46]. Based on the instrumental variable method, model (1) can be re-estimated by two-stage least squares (TSLS):

2 $$First\;stage:\;Childnum_i\;=\;\pi_iZ_v\;+\;\gamma ZX_i\;+\;v_i$$

3 $$Second\;stage:\;L_i\:=\:\beta Childnum_i\:+\:\varphi X_i\:+\:\mu_i$$

In the above formula, *Childnum*_*i*_ is the number of children and *Z*_*v*_ is the instrumental variable and the predicted value of the regression results in the first stage. In order to eliminate the potential heteroscedasticity of the model, the robust standard error is used in this study.

## Results

### Impact of the number of children on older rural women’s longevity

To test the marginal impact of the number of children on women’s longevity, this study used OLS to estimate the correlation parameters between the number of children and the longevity of older rural women. The results in column (1) of Table 2 show a negative correlation between the number of children and the longevity of rural older women. For each additional child, the longevity of rural older women decreases by about 0.555 years (*P* < 0.01).

**Table 2:** The influence of fertility on the longevity of rural older women

	(1)	(2)	(3)	(4)
	Rural female	Rural female	Rural female	Rural female
Number of children	−0.555***			
	(0.151)			
Number of sons		−2.362**		−1.261***
		(1.104)		(0.187)
Number of daughters			0.351*	0.436**
			(0.182)	(0.178)
Marital status	5.705***	5.801***	6.096***	5.504***
	(0.897)	(0.906)	(0.956)	(0.933)
Ethnic group	0.312	0.280	0.843	0.247
	(0.760)	(0.771)	(0.901)	(0.888)
Education status	−3.764***	−4.165***	−4.120***	−4.406***
	(0.932)	(0.933)	(0.969)	(0.943)
Living with family	2.231***	2.205***	2.273***	2.118***
	(0.688)	(0.699)	(0.748)	(0.735)
Hypertension	−3.035***	−3.271***	−3.453***	− 3.231***
	(0.668)	(0.673)	(0.721)	(0.701)
Diabetes	−4.968***	−4.366***	−3.949***	− 4.992***
	(1.317)	(1.338)	(1.393)	(1.365)
Cardiovascular diseases	−0.590	− 0.503	− 0.460	0.055
	(0.717)	(0.727)	(0.768)	(0.748)
Apoplexy	−2.136**	−1.952**	−1.716*	−1.358
	(0.886)	(0.905)	(0.943)	(0.905)
Respiratory diseases	1.994**	1.999**	1.906*	2.057**
	(0.989)	(1.005)	(1.033)	(1.018)
Cancer	−6.997***	−7.183***	−7.050***	−6.692***
	(1.580)	(1.592)	(1.636)	(1.567)
Medical insurance	−1.988**	−1.953**	−1.772*	− 1.617*
	(0.882)	(0.891)	(0.969)	(0.952)
Public older adult insurance	−0.656	− 0.698	− 0.634	−0.967
	(0.676)	(0.685)	(0.718)	(0.708)
Smoking	−13.315*	− 14.618*	− 15.538*	− 15.614**
	(7.883)	(7.942)	(8.030)	(7.679)
Clean energy in the kitchen	0.399	0.406	0.274	0.582
	(0.552)	(0.560)	(0.591)	(0.580)
Middle region	−0.951	−0.852	−1.170	− 0.855
	(0.708)	(0.724)	(0.756)	(0.741)
West region	−1.734**	−1.434**	− 1.553**	−1.840**
	(0.713)	(0.726)	(0.751)	(0.739)
Constant term	95.654***	95.781***	92.450***	92.749***
	(1.396)	(1.622)	(1.455)	(1.475)
Obs.	834	827	754	754
R-squared	0.242	0.231	0.234	0.246

*Note*: Robust standard errors are in parentheses, *** *p* < 0.01, ** *p* < 0.05, * *p* < 0.1; The number of sons and the number of daughters have missing values, resulting in differences in sample size

The results of column (2) of Table 2 show that the number of sons has a significant negative association with the longevity of the rural older women. For every additional son, the longevity of the older adults is reduced by 2.362 years (*P* < 0.01). Column (3) reports the estimated results of the impact of the number of daughters on the longevity of rural older women. The results show that the number of daughters is more conducive to prolonging the mother’s longevity. For each additional daughter, the longevity of the older adults increases by 0.351 years (*P* < 0.1). In column (4), both the number of sons and the number of daughters are included in a model. The number of sons is inversely related to the longevity of rural elderly women, and the number of daughters is changing in the same direction as that of rural elderly women.

Table 3 reports the estimation results after controlling the endogeneity of the model. It can be seen that without controlling the endogeneity of variables, the number of children has a significant negative association with the longevity of rural older women. After controlling for endogeneity, the regression coefficient increases, but it is still significantly negative. This shows that without controlling endogeneity, OLS underestimates the impact of the number of children on the longevity of the rural older women but does not change the basic conclusion. The Cragg Donald Wald F statistic of weak instrumental variable test in the first stage is 24.682, which is higher than the critical value standard of 10 for judging weak instrumental variables, indicating that there is no problem of weak instrumental variables^4^.

**Table 3:** Robustness test: Regression results of two-stage least squares method

Variable	(1)	(2)
	First stage	Second stage
Gender of the first child	−0.270**	
	0.125	
Number of children		−1.645***
		(0.254)
Marital status	0.035	5.616***
	0.202	(0.989)
Ethnic group	−0.075	0.414
	0.172	(0.850)
Education status	0.757***	−5.338***
	0.208	(1.970)
Living with family	−0.280*	2.866***
	0.157	(0.980)
Hypertension	0.550***	−4.196***
	0.150	(1.414)
Diabetes	−0.640**	−3.229
	0.299	(2.077)
Cardiovascular diseases	−0.310*	0.174
	0.162	(1.074)
Apoplexy	−0.043	−1.864*
	0.202	(0.988)
Respiratory diseases	0.198	1.604
	0.224	(1.163)
Cancer	0.425	−7.870***
	0.355	(1.973)
Medical insurance	0.073	−2.271**
	0.201	(0.995)
Public older adult insurance	−0.049	−0.572
	0.153	(0.755)
Smoking	2.058	−18.271*
	1.776	(10.028)
Clean energy in the kitchen	−0.035	0.392
	0.125	(0.619)
Middle region	0.204	−1.254
	0.161	(0.886)
West region	−0.098	− 1.645**
	0.162	(0.832)
Constant term	3.710***	87.842***
	0.300	(8.212)
Obs.	827	827
R-squared	0.158	0.156
Model test	Cragg-Donald Wald F statistic: 24.682, *p* = 0.000	

*Note*: Robust standard errors are in parentheses, *** *p* < 0.01, ** *p* < 0.05, * *p* < 0.1; The number of sons and the number of daughters have missing values, resulting in differences in sample size

### Impact of gender differences and other reproductive behaviours on older rural women’s longevity

To further verify whether the research findings for age at first birth, age at last birth, and other reproductive behaviours have an impact on longevity in China, and in consideration of Chinese fertility culture, where greater value is placed on sons, this study selected having a son, having a daughter, having both sons and daughters, age at first birth, and age at last birth as explanatory variables to continue to examine the effects of other reproductive behaviours on older rural women’s longevity.

Columns (1) and (2) of Table 4 respectively report the estimated results of the impact of whether to have a son and whether to have a daughter on the longevity of rural female women. The results show that having a son has a significant negative association with the longevity of rural older women, but the association between having a daughter and the longevity of rural older women is not significant. The estimated results in column (3) of Table 4 show that the association between having both sons and daughters and the longevity of rural older women is also not significant.

**Table 4:** Impact of children’s gender differences and other reproductive behaviours on older rural women’s longevity

	(1)	(2)	(3)	(4)	(5)	(6)
Having a son	−2.342**					
	(1.095)					
Having a daughter		0.312				
		(0.843)				
Having sons and daughters			−0.526			
			(0.736)			
Age at first birth				0.251***		0.135**
				(0.055)		(0.057)
Age at last birth					0.263***	0.222***
					(0.043)	(0.045)
Marital status	5.767***	5.810***	5.786***	5.996***	5.276***	4.890***
	(0.905)	(0.909)	(0.908)	(0.965)	(0.971)	(0.926)
Ethnic group	0.284	0.337	0.370	0.696	0.613	0.071
	(0.770)	(0.773)	(0.772)	(0.936)	(0.941)	(0.915)
Education status	−4.143***	−4.249***	−4.256***	−3.928***	−4.046***	−4.269***
	(0.933)	(0.935)	(0.935)	(0.974)	(0.967)	(0.933)
Living with family	2.262***	2.227***	2.211***	2.170***	2.667***	2.370***
	(0.696)	(0.701)	(0.701)	(0.766)	(0.770)	(0.739)
Hypertension	−3.263***	− 3.341***	− 3.301***	− 3.045***	− 3.319***	−3.037***
	(0.672)	(0.675)	(0.675)	(0.730)	(0.730)	(0.699)
Diabetes	−4.552***	−4.398***	− 4.434***	− 4.444***	−3.716***	−4.484***
	(1.324)	(1.343)	(1.342)	(1.422)	(1.416)	(1.365)
Cardiovascular diseases	−0.536	−0.404	−0.464	−0.389	−0.302	−0.067
	(0.726)	(0.731)	(0.730)	(0.786)	(0.784)	(0.749)
Apoplexy	−2.027**	−1.930**	−1.903**	−1.692*	−1.661*	−1.261
	(0.902)	(0.908)	(0.908)	(0.955)	(0.959)	(0.912)
Respiratory diseases	2.019**	1.928*	1.946*	1.869*	1.812*	1.939*
	(1.005)	(1.008)	(1.008)	(1.046)	(1.038)	(1.002)
Cancer	−7.180***	−7.245***	−7.223***	−7.088***	−6.664***	−6.414***
	(1.591)	(1.596)	(1.596)	(1.633)	(1.621)	(1.541)
Medical insurance	−1.969**	−2.024**	−2.047**	− 1.565	− 1.565	− 1.624*
	(0.890)	(0.893)	(0.892)	(0.983)	(0.981)	(0.944)
Public older adult Insurance	−0.686	−0.672	−0.709	−0.840	−0.583	−0.833
	(0.684)	(0.688)	(0.687)	(0.727)	(0.731)	(0.707)
Smoking	−14.645*	−14.648*	−14.530*	− 14.003*	−15.221*	− 14.301*
	(7.937)	(7.964)	(7.963)	(7.992)	(7.920)	(7.529)
Clean energy in the kitchen	0.428	0.404	0.414	0.390	0.555	0.869
	(0.558)	(0.562)	(0.561)	(0.600)	(0.603)	(0.582)
Middle region	−0.842	−1.046	−1.021	−0.586	−1.237	− 0.992
	(0.723)	(0.720)	(0.721)	(0.763)	(0.772)	(0.748)
West region	−1.438**	−1.614**	− 1.558**	−1.481*	− 1.929**	−2.061***
	(0.725)	(0.724)	(0.726)	(0.760)	(0.758)	(0.734)
_cons	95.751***	93.441***	94.180***	87.097***	83.473***	81.904***
	(1.615)	(1.523)	(1.457)	(1.932)	(2.103)	(2.186)
Obs.	829	827	827	722	704	704
R-squared	0.234	0.227	0.227	0.248	0.268	0.284

*Note*: Robust standard errors are in parentheses, *** *p* < 0.01, ** *p* < 0.05, * *p* < 0.1

Columns (4) and (5) of Table 4 respectively report the estimated results of the impact of the age at first birth and the age at last birth on the longevity of rural older women. The results show that the older the age at first birth, the more conducive it is to the extension of longevity of the older women. With every increase by 1 year in the age at first birth, the longevity will increase by 0.251 years (*P* < 0.01). The older the age at last birth was, the longer the longevity of the female (*P* < 0.01). If the age increased by 1 year, the longevity would be extended by 0.263 years. In column (6), the age at first birth and the age at last birth are included together in the model. The age at first birth and the age at last birth are still significantly positively correlated with the longevity of rural older women.

### Influence of the number of children on older adults’ longevity in urban and rural areas

There are great economic, social, and cultural differences between urban and rural areas in China. To further test whether there was a difference between urban and rural areas regarding the impact of the number of children on longevity, the whole sample, older urban adults and older rural men, were included in the analysis. By comparison, we further examine whether the trade-off between fertility and longevity is prevalent among older adults in China. Column (1) of Table 5 reports the impact of the number of children on the longevity of the entire sample. The results are consistent with the basic regression results; the more children an older adult has, the lower their longevity.

**Table 5:** Urban and rural differences between the impact of number of children on older adults’ longevity

	(1)	(2)	(3)	(4)	(5)	(6)
	Full sample	Urban	Rural	Urban female	Urban male	Rural male
Number of Children	−0.229**	−0.328	−0.241**	−0.195	−0.577	0.156
	(0.102)	(0.432)	(0.106)	(0.713)	(0.619)	(0.147)
Marital status	5.188***	4.650***	5.243***	−1.255	5.632***	5.083***
	(0.501)	(1.663)	(0.531)	(4.079)	(1.851)	(0.654)
Ethnic group	0.099	0.613	−0.047	2.424	0.968	−0.439
	(0.549)	(3.111)	(0.559)	(5.658)	(3.832)	(0.812)
Educated	−2.619***	−1.885	−2.616***	−4.536	− 1.457	−2.060***
	(0.483)	(1.916)	(0.505)	(3.205)	(2.643)	(0.589)
Living with family	2.263***	1.545	2.303***	1.126	2.149	2.300***
	(0.451)	(1.731)	(0.472)	(3.565)	(2.175)	(0.637)
Hypertension	−2.646***	−4.836***	− 2.400***	−1.346	−6.406***	−1.401**
	(0.459)	(1.760)	(0.479)	(3.423)	(2.114)	(0.677)
Diabetes	−3.739***	−4.712	−3.778***	− 4.107	−5.043	−2.919**
	(0.869)	(2.923)	(0.917)	(5.378)	(3.558)	(1.257)
Cardiovascular diseases	−0.248	0.371	−0.147	−4.924	2.455	0.376
	(0.500)	(1.767)	(0.526)	(3.233)	(2.339)	(0.760)
Apoplexy	−2.060***	−2.681	−1.912***	−8.875**	−1.014	−1.754**
	(0.587)	(1.843)	(0.622)	(3.467)	(2.360)	(0.859)
Respiratory diseases	−0.777	−3.007	−0.458	−4.581	−2.238	−1.783**
	(0.567)	(1.828)	(0.602)	(2.986)	(2.374)	(0.748)
Cancer	−3.382***	−1.421	−3.598***	3.739	−2.052	−2.787***
	(0.652)	(1.885)	(0.707)	(4.084)	(2.190)	(0.767)
Medical insurance	−1.050	0.281	−1.294*	0.027	−0.127	−0.177
	(0.641)	(2.091)	(0.677)	(3.089)	(3.125)	(1.062)
Public older adult insurance	−0.650	− 0.815	−0.652	1.731	−3.979	−0.627
	(0.464)	(2.175)	(0.476)	(3.081)	(3.800)	(0.660)
Smoking	−6.472	−5.765	−11.017	–	−1.094	–
	(5.517)	(8.984)	(7.752)	–	(9.351)	–
Clean energy in the kitchen	1.014***	4.186**	0.802**	7.432**	3.056	1.442**
	(0.391)	(1.817)	(0.404)	(3.312)	(2.281)	(0.583)
Middle region	−1.265***	−5.313***	−0.899*	−3.927	−7.144***	−0.867
	(0.485)	(1.810)	(0.507)	(2.869)	(2.594)	(0.721)
West region	−0.939*	−1.383	− 0.926*	−1.774	−0.218	− 0.170
	(0.486)	(1.820)	(0.507)	(3.304)	(2.484)	(0.712)
Male	−1.967***	−2.490	−1.950***			
	(0.455)	(1.759)	(0.474)			
Urban	0.191					
	(0.692)					
Constant term	93.475***	93.126***	93.567***	91.455***	96.428***	88.053***
	(0.939)	(3.267)	(0.987)	(4.039)	(7.647)	(1.450)
Obs.	1678	148	1530	86	62	696
R-squared	0.293	0.423	0.285	0.470	0.391	0.233

*Note*: Robust standard errors are in parentheses; *** *p* < 0.01, ** *p* < 0.05, * *p* < 0.1

Columns (2) and (3) of Table 5 respectively report the estimated impact of the number of children on the longevity of the urban and rural older women. The results show that the number of children negatively correlates with the longevity of the rural older women, but the association with the longevity of the urban older women is not significant. Every increase in the number of children will reduce the rural older woman’s longevity by 0.241 years (*P* < 0.05).

Columns (2) and (3) of Table 5 report the estimated impact of the number of children on the longevity of older urban and rural adults, respectively. The number of children negatively correlates with the longevity of older urban and rural adults, but the correlation with older urban adults is not significant. For each additional child, the longevity of an older rural adult will decrease by 0.241 years (*p* < 0.05). The estimated results in columns (4), (5), and (6) of Table 5 show that the number of children has no significant association with the longevity of urban older women, urban older men, and rural older men.

## Discussion

The results show that the number of children, the number of sons, and having a son have a significant negative association with the longevity of rural older women and that the trade-off between fertility and longevity is mainly reflected in older women in rural China. The number of daughters, age at first birth and age at last birth have a significant positive association with the longevity of rural older women. Furthermore, the impact of fertility on the longevity of older adults differs between urban and rural areas. Next, we will discuss these results in more detail.

### The higher the number of births, the lower rural women’s longevity

In addition to the physiological mechanisms underlying the effect of fertility on longevity described by DST, there are also socio-economic factors that influence the longevity of rural Chinese women, as suggested by Dribe [5], and the Chinese context should, therefore, be considered. The fertility period of these older adults was mostly before 1980, when China had not yet started implementing strict family planning policies, and contraceptives had not yet been popularised. There was also a widespread perception of ‘more children, more blessings’. Before the 1980s, China’s low productivity and frequent natural disasters led to recurrent famines in rural areas. Women often faced hunger and malnutrition during the perinatal period. At that time, contraceptive methods were scarce, and the more children were born, the more adverse it was to women’s health in rural areas, ultimately affecting their longevity. According to Grossman’s [47] health production theory, health is not only a consumer good but an investment good. Apart from times of illness, one part of a person’s life is used for work and the production of goods, and the other for leisure. Health can be attained only when work and leisure times are equal. However, before China’s reform, more children meant that women in rural areas had to spend more time on childcare and housework. Children would occupy mothers’ leisure time and more family resources, resulting in mothers not having much time for health attainment.

### Having a son lowers maternal longevity, and having a daughter raises it

The negative influence of child gender structure on mothers’ longevity is mainly through biological and social factors [31, 34]. From a biological viewpoint, the physiological cost of having a son is higher than that of having a daughter [30]. A son grows faster in the womb and has a higher birth weight [48, 49]. Mothers also need to consume more energy during pregnancy [50], which negatively impacts their psychology and physiology. From a social viewpoint, especially in Chinese rural areas, investment in education for girls is less than for boys, and girls begin to earn labour subsidies for families as children. In East Asian culture, women’s marriages can bring cash gifts to parents via dowries, while men’s marriages cannot. Further, the high cost of education and marriage leads to less participation in housework and later participation in social work, leading to the high costs of bearing sons [34, 49]. Previous studies found that sons consumed more family resources (mainly in the form of food), and for families who experience food shortages, sons have a negative impact on mothers’ longevity [32, 36]. In addition, considering the gender inequality in housework and mental health, having a daughter may be more conducive to prolonging mothers’ longevity [30].

### The number of births does not affect the longevity of older urban women and rural men

Dribe [5] found that socio-economic status is an important channel that affects longevity. The socio-economic status of older urban adults is higher than older rural adults in China. Compared to rural women, urban women are less affected by the economic constraints of fertility. They can supplement their nutrition during the perinatal period in a timelier manner to make up for the decline in longevity caused by fertility. Second, compared to rural women, urban women benefit from the maternity insurance system, prenatal examinations, and postpartum rehabilitation, and many high-quality medical resources are concentrated in urban areas. Thus, urban women can enjoy high-quality public health and medical services and can discover and treat diseases during the fertility process in a timelier fashion. Third, the distribution of housework is more equal in urban than in rural areas, with men sharing a portion of the child-rearing chores. Thus, when compared to rural women, why does the number of births have no significant effect on the longevity of rural older men? First, according to DST, fertility only takes a physical toll on women. Second, in terms of parenting, the division of labour—that is, the ‘male dominates the outside, and the female dominates the inside’—is implemented in rural areas, and women do most of the housework. The greater the number of children born, the greater the burden of domestic work required by rural women, and thus the more significant the negative impact on health and longevity.

### Having children later in life helps to increase rural women’s longevity

This finding differs from those of previous research. For example, Dribe [5], Fuster [37], and Lockhart, Martin, Johnson, Shirtcliff, and Poon [22] did not find a significant effect of age at first birth on mortality in old age in their studies that were based on Swedish, Spanish, and Georgian samples. This result may be closely related to the tradition of early marriage and childbearing in rural China. Under China’s 1950 marriage law, the legal age for marriage is no earlier than 20 years for men and 18 years for women. In rural areas, women tend to marry and have children at a younger age, which is not conducive to their health because they are not yet fully developed. With the gradual increase in fertility age, women enter a relatively mature fertility period conducive to good health and increased longevity.

First, this finding may be related to the birth spacing of rural women. The older the age at the last birth, the longer the interval between births, which avoids the need for women to raise multiple children in a short period, reduces the burden of care on women, and contributes to their improved health status. Second, the older the age at which a woman gives birth also reflects a higher level of fitness and is an act of self-selection, with the last birth delaying the onset of menopause and slowing down women’s ageing [51, 52]. The later the age at the last birth, the later is the onset of menopause. According to Lockhart, Martin, Johnson, Shirtcliff, and Poon [22], the later the onset of menopause, the slower the ageing, and thus the increase in longevity. Of course, having the last child at an older age also has a spiritual impact on health, as the need to raise the child leads to a high degree of dutifulness, and highly dutiful individuals usually live longer [52].

### Limitations

Although this study confirms a trade-off relationship between fertility and longevity in older women in rural China, there are still some limitations.

First, the dependent variable is limited. Because the death age of the sample used in this study is greater than 65 years old, it is impossible to observe the longevity and fertility behaviour of people whose death age is less than 65 years old, which may overestimate the impact of fertility on longevity.

Second, the samples we used had given birth to children, and those women who died during pregnancy or childbirth were excluded from the study, which may lead to a certain degree of sample selection bias.

Third, we only choose the gender of the first child as the instrumental variable of the number of children, while other reproductive behaviours, such as the age at the first birth and the age at the last birth, may also have endogenous problems. Due to the failure to find suitable instrumental variables for further tests, the estimation results of OLS may be biased.

Fourth, the gender of the first child not only indirectly affects the mother’s longevity through the number of children, but also may directly affect the mother’s longevity. Fertility behaviours themselves will lead to the loss of mother’s health, so the gender of the first child may not fully meet the exclusive conditions.

Fifth, Table 5 compares whether there are significant urban-rural differences in the impact of fertility on the longevity of the elderly. Because there are many missing values of explanatory variables, the sample number of urban men and urban women is less. In urban male or urban female samples, no significant correlation between the number of children and longevity was found. However, a lack of statistical significance does not mean that the effect does not exist. Small samples do miss an effect that does exist as a whole. When the sample size is small, using the 10% level threshold will increase the probability of type I error and lead to the bias of estimation results. The trade-off between fertility and longevity may exist not only in rural women, but also in urban women. In the samples used in this study, the number of rural samples is large, and the number of urban samples is smaller in comparison. Therefore, there may be some bias in the estimation results of urban samples. In the future, we hope to obtain a more extensive data set of death information in an urban sample of older people and continue to verify whether there is a trade-off between fertility and longevity.

## Conclusion

Based on data from the 2014–2018 CLHLS, this study empirically analysed the relationship between the number of children and the longevity of older rural women using the OLS method. It found that a higher number of children born was less conducive to the extension of older rural women’s longevity. For each additional child, older rural women’s longevity was reduced by 0.555 years. Similarly, a higher number of sons was not conducive to a longer longevity among older rural women, yet a higher number of daughters was. A trade-off between fertility and longevity was found for older rural women in China but not for older urban adults or older rural men. The study also found that the older the age at first and last births, the higher the increase in the older rural women’s longevity.

The current findings suggest that to better protect women who give birth, especially rural women in developing countries, a system of subsidised hospital births should be implemented in the future. This includes providing free basic health care services to pregnant women throughout pregnancy and childbirth, compensating for the health declines from fertility in rural women of fertility age, and improving the fertility of couples of fertility age. Further, this system should continue to attend to the sex ratio of the birth population and strictly prohibit gender discrimination. It should also advocate for a healthy pregnancy and improve the screening rate, early diagnosis, and treatment rate of common diseases among women through public education and guidance. Physical intervention programmes should be developed and implemented for women, especially rural women, to popularise knowledge of contraception, birth control, and reproductive health. Rural residents should be guided to have children in a planned manner to improve the national family planning technical service policy and increase the protection of family planning technical services for subsequent births.

## Data Availability

The data that support the findings of this study are openly available from the Peking University Open Research Data of the CLHLS. [10.18170/DVN/UWS2LR].

## Abbreviations

CLHLS: Chinese Longitudinal Healthy Longevity Survey
DST: Disposable soma theory
OLS: Ordinary least squares

## Footnotes

1 The birth experience of all samples was before 1980, which eliminates the influence of China’s family planning policy on birth behaviour.

2 The results of the Skewness/Kurtosis tests for Normality cannot reject the assumption of normal distribution.

3 It is worth noting that the gender of a child can affect the longevity of rural older women directly and indirectly through the number of children. it might not guarantee that the instrumental variable met the exclusion restriction. We will discuss this deficiency in the limitations.

4 Because we only used one instrumental variable, and the number of endogenous variables and instrumental variables were exactly the same, we could not carry out the overidentification test, so we did not report the results of the overidentification test.
